# Virulence and extended-spectrum β-lactamase encoding genes in *Escherichia coli* recovered from chicken meat intended for hospitalized human consumption

**DOI:** 10.14202/vetworld.2017.1281-1285

**Published:** 2017-10-28

**Authors:** Gamal A. Younis, Rasha M. Elkenany, Mohamed A. Fouda, Noura F. Mostafa

**Affiliations:** 1 Department of Bacteriology, Mycology and Immunology, Faculty of Veterinary Medicine, Mansoura University, Egypt; 2 Department of Nutrition, Gastroenterology Surgery Center, Mansoura University, Egypt

**Keywords:** *bla*_OXA_, *bla*_TEM_, *eaeA*, *Escherichia coli*, extended-spectrum β-lactamases, *stx1*, *stx2*

## Abstract

**Aim:**

This study describes the prevalence of *Escherichia coli* in frozen chicken meat intended for human consumption with emphasis on their virulence determinants through detection of the virulence genes and recognition of the extended-spectrum β-lactamase (ESBL) encoding genes (*bla*_OXA_ and *bla*_TEM_ genes).

**Materials and Methods:**

A total of 120 frozen chicken meat samples were investigated for isolation of *E. coli*. All isolates were subjected to biochemical and serological tests. Eight serotypes isolated from samples were analyzed for the presence of various virulence genes (*stx1, stx2*, and *eae A* genes) using multiplex polymerase chain reaction (PCR) technique. Moreover, the strains were evaluated for the ESBL encoding genes (*bla*_TEM_ and *bla*_OXA_).

**Results:**

Overall, 11.66% (14/120) chicken meat samples carried *E. coli* according to cultural and biochemical properties. The most predominant serotypes were O78 and O128: H2 (21.5%, each), followed by O121: H7 and O44: H18. Molecular method detected that 2 strains (25%) harbored *stx1*, 3 strains (37.5%) *stx2*, and 3 strains (37.5%) both *stx1* and *stx2*, while 1 (12.5%) strain carried *eae A* gene. Particularly, only O26 serotype had all tested virulence genes (*stx1, stx2, and eae A*). The results revealed that all examined 8 serotypes were Shiga toxin-producing *E. coli* (STEC). The ESBL encoding genes (*bla*_TEM_ and *bla*_OXA_) of STEC were detected in 4 (50%) isolates by multiplex PCR. The overall incidence of *bla*_TEM_ and *bla*_OXA_ genes was 3 (37.5%) and 2 (25%) isolates.

**Conclusion:**

The present study indicates the prevalence of virulent and ESBL-producing *E. coli* in frozen chicken meat intended for hospitalized human consumption due to poor hygienic measures and irregular use of antibiotics. Therefore, the basic instructions regarding good hygienic measures should be adapted to limit public health hazard.

## Introduction

Food represents a possible source of pathogenic- and antibiotic-resistant *Escherichia coli* strains [1]. Infections due to pathogenic *E. coli* may be restricted to the mucosal surfaces or distributed throughout the body [2]. Various intestinal and extraintestinal diseases can be caused by *E. coli* isolates harbored virulence genes. Intestinal pathological types are enterotoxigenic *E. coli* (ETEC), enteropathogenic *E. coli* (EPEC), enteroaggregative *E. coli* (EAEC), enteroinvasive *E. coli* (EIEC), diffusely adherent *E. coli* (DAEC), and Shiga toxin-producing *E. coli* (STEC). *E. coli* strains that provide Shiga toxins are called STEC, vero cytotoxin-producing *E. coli*, or enterohemorrhagic *E. coli* (EHEC) [3,4]. Mild-to-severe diarrhea and colitis are resulted from strains of these pathological types (ETEC, EPEC, EAEC, EIEC, and DAEC) [5]. Whatever, STEC is associated with a wide range of human diseases such as bloody diarrhea, hemorrhagic colitis (HC), and hemolytic-uremic syndrome (HUS) [5]. The Shiga toxins either 1 (*stx1*) or 2 (*stx2*) interfere the binding of aminoacyl tRNA to the ribosomes and preventing the protein synthesis resulting in depurinating specific residues of the host cell ribosomes after internalization [6]. The biological activities of *stx1* and *stx2*, involving cytotoxicity to Vero and HeLa cells, are similar, but the immunological properties are different [7].

The members of β-lactam antimicrobial agents involve penicillin, cephalosporin, clavams, and cephamycins which have a β-lactam. The hydrolyzing of β-lactam ring by β-lactamases is responsible for the inactivation of β-lactam antibiotics. The most commonly identified β-lactamases are TEM-, SHV-, OXA-, CMY-, and CTX-M-β-lactamases in Gram-negative bacteria [8]. The *bla*_OXA_ gene as antibiotic-resistant gene encodes a carbapenem-hydrolyzing class D-lactamase [9]. The occurrence of infections with extended-spectrum β-lactamase (ESBL)-producing *E. coli* in humans is increased as a result of intestinal carriage of ESBL-producing bacteria in food animals as well as infectivity of retail meat [10]. Consequently, the transmission of ESBL-producing *E. coli* to humans through consumption of chicken has become a public health hazard [11].

Therefore, the purpose of this study was to investigate the prevalence of *E. coli* and their serotypes in chicken meat intended for hospitalized human consumption with emphasis on their virulence determinants through the finding of virulence factors (*stx1, stx2*, and *eae* genes) and recognition of ESBL encoding genes (*bla*_OXA_ and *bla*_TEM_ genes) using multiplex polymerase chain reaction (PCR).

## Materials and Methods

### Ethical approval

In this investigation, we did not use live animals; therefore, ethical approval was not essential. Chicken meat samples were obtained from Gastroenterology Surgery Center (GEC).

### Sampling

A total of 120 frozen chicken meat samples were obtained during October 2015 from GEC, Mansoura University, Egypt. The samples were subjected to bacteriological analysis.

### Bacteriological analysis

A sample of 25 g from each chicken meat sample was homogenized in 225 ml of bacteriological peptone water and incubated at 37°C for 18-24 h. Then, after incubation, 0.1 ml from peptone water was cultured onto MacConkey’s agar and Eosin methylene blue (EMB) agar (Oxoid Ltd., England) and incubated at 37°C for 24 h. The colonies with pink color on MacConkey’s agar and green metallic sheen on EMB agar were considered as *E. coli*. In addition, it has known that some *E. coli* show purple color with or without metallic sheen on EMB. The following biochemical tests were applied for the identification of suspected colonies: Triple sugar iron agar, citrate utilization, urease production, indole, methyl red, Voges-Proskauer tests, and motility. The diagnostic *E. coli* antisera sets (DENKA SEIKEN Co., Japan) were used for serological identification of enteropathogenic types of *E. coli* isolates depending on O and H antigens [12].

### Molecular detection of virulence genes and ESBL encoding genes

Eight different *E. coli* serotypes were used for the detection of virulence factors involving Shiga toxins (*stx1* and *stx2*) and intimin (*eaeA*) genes as well as ESBL-encoding genes (*bla*_TEM_ and *bla*_OXA_) by multiplex PCR. DNA extraction was performed using QIA amp kit [13]. The amplification reaction was performed using specific primers and profiles as shown in Tables-1 and 2 [14-16]. The analysis of PCR products was applied by 2% agarose gel electrophoresis (AppliChem, Germany, GmbH) in 1× TBE buffer stained with ethidium bromide, followed by visualization on an ultraviolet transilluminator.

**Table-1:** Primer sequences of *E. coli* virulence genes and extendedspectrum β-lactamase encoding genes.

Target gene	Oligonucleotide sequence	Product size (bp)	References
*stx1* (F)	5′ ACACTGGATGATCTCAGTGG ′3	614	[14]
*stx1* (R)	5′ CTGAATCCCCCTCCATTATG ′3		
*stx2* (F)	5′ CCATGACAACGGACAGCAGTT ′3	779	
*stx2* (R)	5′ CCTGTCAACTGAGCAGCACTTTG ′3		
*eaeA* (F)	5′ GTGGCGAATACTGGCGAGACT ′3	890	[15]
*eaeA* (R)	5′ CCCCATTCTTTTTCACCGTCG ′3		
*bla*_OXA_ (F)	5′ GGCACCAGATTCAACTTTCAAG ′3	564	[16]
*bla*_OXA_ (R)	5′ GACCCCAAGTTTCCTGTAAGTG ′3		
*bla*_TEM_ (F)	5′ CATTTCCGTGTCGCCCTTATTC ′3	800	
*bla*_TEM_ (R)	5′ CGTTCATCCATAGTTGCCTGAC ′3		

**Table-2:** Cycling conditions of the different primers during PCR.

Target gene	Primary denaturation	Secondary denaturation	Annealing	Extension	Final extension
*stx1*	95°C	95°C	58°C	72°C	72°C
	3 min	20 s	20 s	1.5 min	5 min
*stx2*	95°C	95°C	58°C	72°C
	3 min	20 s	20 s	1.5 min
*eaeA*	95°C	95°C	58°C	72°C
	3 min	20 s	20 s	1.5 min
*bla*_OXA_ and *bla*_TEM_	94°C	94°C	61°C	72°C	72°C
	10 min	30 s	35 s	1 min	1 min

PCR=Polymerase chain reaction

## Results and Discussion

Food of chicken origin has been a source of virulent and antimicrobial-resistant *E. coli* strains that responsible for a serious public health worldwide causing food poisoning in humans [1]. In this work, 14 (11.66%) of 120 chicken meat samples carried *E. coli* according to cultural and biochemical properties. This result indicates relatively low prevalence rate of *E. coli* in chicken meat intended for human consumption in GEC. A similar observation was recorded by other researchers who detected 11.1% and 15.8% of *E. coli* in chicken meat [17,18], respectively. In contrast, Rashid *et al*. [19], Adeyanju and Ishola [20], and Park *et al*. [21] found 40%, 43.4%, and 75.9% of *E. coli* in poultry meat, respectively. Whatever, *E. coli* should be lower than the infective dose in chicken meat, particularly STEC to be considered fit for human consumption. Therefore, the preparation of healthy chicken meat is necessary for public health. However, the contamination of bird carcasses can occur following slaughter and dressing with predominantly enteric bacteria, including *E. coli*, coming from the skin, hair, feathers, gastrointestinal tract, and the environment at the slaughtering facilities [22].

Serological test of recovered strains identified eight different *E. coli* serotypes (Table-3). Among identified serotypes, the most predominant serotypes were O78 and O128: H2 (21.5%, each), followed by O121: H7 and O44: H18 (14.3%, each) in chicken meat. This result is consistent with the previous study that detected these serotypes in *E. coli* of avian origin [23]. In addition, our results revealed three subgroups that were EPEC (7, 50%), followed by EHEC (4, 28.5%) and ETEC (3, 21.5%). EPEC was the most common subgroup compared to other researchers who detected only 4% of EPEC in chicken meat [19] and a variable quantity of EPEC [24]. Nearly similar, Momtaz and Jamshidi [25] identified AEEC (34.93%) and EHEC (21.23%) subgroups among *E. coli* isolates from chicken meat.

**Table-3:** Prevalence and different serotypes of *E. coli* recovered from chicken meat.

Serotypes	Number of strains	Frequency distribution (%)
O44:H18	2	14.30
O78	3	21.50
O2:H6	1	7.10
O153:H2	1	7.10
Total	7	50
O121:H7	2	14.30
O91:H21	1	7.10
O26:H11	1	7.10
Total	4	28.50
O128:H2	3	21.50
Overall total	14	11.66

**Figure-1:**
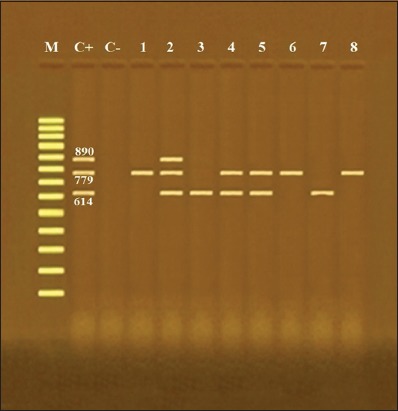
Agarose gel electrophoresis of multiplex polymerase chain reaction of *stx1* (614 bp), *stx2* (779 bp), and *eaeA* (890 bp) genes for characterization of different *Escherichia coli* serotypes. Lane M: 100 bp ladder as molecular size DNA marker, Lane C+: Control positive *E. coli* for *stx1*, *stx2*, and eaeA genes, Lane C-: Control negative, Lanes 1 (O2), 6 (O121), and 8 (O153): Positive *E. coli* strains for *stx2* gene only, Lanes 3 (O44) and 7 (O128): Positive *E. coli* strains for *stx1* gene only, Lane 2 (O26): Positive *E. coli* strain for *stx1*, *stx2*, and eaeA genes, and Lanes 4 (O78) and 5 (O91): Positive *E. coli* strains for *stx1* and *stx2* genes.

According to multiplex PCR assay, different virulence factors (*stx1, stx2*, and *eaeA* genes) to eight different serotypes were identified (Table-4) (Figure-1). Overall, 2 (25%) strains harbored *stx1*, 3 (37.5%) *stx2*, and 3 (37.5%) both *stx1* and *stx2*, while 1 (12.5%) strain carried *eae A* gene. Particularly, only O26 serotype had all tested virulence genes (*stx1, stx2*, and *eaeA*). From these results, all examined 8 (100%) serotypes were STEC. In another study, *E. coli* isolates had *stx1 (*10.5%), *stx2 (*7%), both *stx1* and *stx2 (*1.5%), and *eaeA* (8%) virulence genes [19] that were lower than this study. Other investigators detected both *stx1* and *eae* genes in all strains, but no strains had the *stx2* [21]. However, other researchers detected the low prevalence of STEC in chicken meat [1,19].

**Table-4:** Occurrence of virulence and extended-spectrum β-lactamase encoding genes in different *E. coli* serotypes recovered from chicken meat.

Sample number	Serotypes	Virulence genes					β-lactamase genes	
	
		*stx1*	*stx2*	*stx1* and *stx2*	*eae A*	*stx1*, *stx2* and *eae A*	*bla*_TEM_	*bla*_OXA_
1	O121:H7	-	+	-	-	-	-	-
2	O44:H18	+	-	-	-	-	-	-
3	O78	-	-	+	-	-	+	+
4	O128:H2	+	-	-	-	-	-	+
5	O153:H2	-	+	-	-	-	-	-
6	O91:H21	-	-	+	-	-	+	-
7	O26:H11	-	-	+	+	+	+	-
8	O2:H6	-	+	-	-	-	-	-
Total (%)	8	2 (25)	3 (37.5)	3 (37.5)	1 (12.5)	1 (12.5)	3 (37.5)	2 (25)

*Stx1* = Shiga toxin 1 gene of *E. coli*, *Stx2* = Shiga toxin 2 gene of *E. coli, eae A*=Intimin gene of *E. coli,* *bla*_TEM_ and *bla*_OXA_=Extended-spectrum β-lactamase-resistant genes of *E. coli=Escherichia coli*

There are serious diseases resulted from STEC strains in humans and animals. The severity of such diseases is related to the type and amount of the produced Shiga toxin [7]. Consequently, the extensive studies have been applied to the type of Shiga toxin formed by STEC recovered from human infections [26]. Besides Shiga toxin virulence genes, Law [7] has detected the *eaeA* gene. In this study, the virulence genes concerning *stx1, stx2*, and *eaeA* were detected in *E. coli* strains recovered from frozen chicken meat. Thus, serious illness such as HUS and HC can occur from the ingestion of raw or undercooked chicken meats in humans.

**Figure-2:**
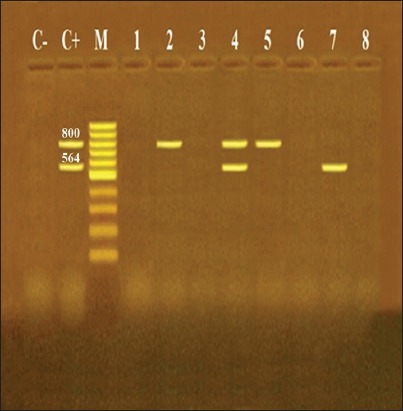
Agarose gel electrophoresis of multiplex polymerase chain reaction of *bla*_OXA_ (564 bp) and *bla*_TEM_ (800 bp) as antibiotic resistance genes of different *E. coli* serotypes. Lane M: 100 bp ladder as molecular size DNA marker, Lane C+: Control positive for *bla*_TEM_ and *bla*_OXA_ genes, Lane C-: Control negative, Lanes 2 (O26) and 5 (O91): Positive *E. coli* strains for *bla*_TEM_ gene only, Lane 7 (O128): Positive *E. coli* strain for *bla*_OXA_ gene only, Lane 4 (O78): Positive *E.coli* strain for both *bla*_OXA_ and *bla*_TEM_ genes, and Lanes 1 (O2), 3 (O44), 6 (O121), and 8 (O153): Negative *E. coli* strains for *bla*_OXA_ and *bla*_TEM_ genes.

The multiplex PCR assay is an efficient and rapid method for identification of extended-spectrum β-lactamase (ESBL) in *E. coli* isolates. In this work, the ESBL encoding genes (*bla*_TEM_ and *bla*_OXA_) of STEC were detected in 4 (50%) isolates by multiplex PCR (Table-4) (Figure-2). Similarly, 49% of the *E. coli* isolates were ESBL producers isolated from chicken meat by Mbanga *et al*. [27]. In contrast, another study reported 94% of ESBL-producing *E. coli* isolates recovered from retail meat samples [11]. From these results, the overall occurrence of *bla*_TEM_ and *bla*_OXA_ genes was 3 (37.5%) and 2 (25%) isolates, respectively. There was one isolate harbored both *bla*_TEM_ and *bla*_OXA_ genes. The *bla*_TEM_ gene (37.5%) was the predominant one among the isolated strains. The previous studies support our findings that detected *bla*_TEM_ gene as the most recurrent β-lactamase reliable for β-lactam resistance [28,29]. Furthermore, other investigators detected *bla*_TEM_ with the absence of *bla*_OXA_ genes in ESBL-producing isolates from chicken meat [27]. It seems that the irregular use of β-lactam antibiotics as broad-spectrum antibacterial agents in poultry farms is correlated to the emergence of ESBL-producing isolates in Egypt. Consequently, the isolated STEC strains from chicken meat are a potential reservoir of β-lactamase genes.

## Conclusion

Frozen chicken meat may be notable hazards to humans because they may carry STEC- and ESBL-producing *E. coli* due to poor hygienic practices. Therefore, it is necessary to improve hygienic measures during the manipulation of meat products to limit public health issue. Furthermore, coordinated measures are essential to decrease or prevent the risks caused by *E. coli* at different stages in the food chain. In addition, the problems related to infections by STEC- and ESBL-producing strains can be overcome through appropriate strategy for infection control in hospital settings.

## Authors’ Contributions

GAY designed and planned this research work. NFM collected the samples and executed the isolation, biochemical, serological, and molecular characterization work of all isolates. RME and MAF analyzed the data and monitored the isolation, biochemical, serological, and molecular characterization. All authors contributed equally in preparation and revision of the manuscript. All authors read and approved the final manuscript.
